# Clinical and molecular data from 61 Brazilian cases of Congenital Hyperinsulinemic Hypoglycemia

**DOI:** 10.1186/1758-5996-7-5

**Published:** 2015-02-18

**Authors:** Raphael Del Roio Liberatore, Priscila Manzini Ramos, Gil Guerra, Thais Della Manna, Ivani Novato Silva, Carlos Eduardo Martinelli

**Affiliations:** Ribeirão Preto Medical School, University of São Paulo, Rua Elzira Sammarco Palma, 400/43, Ribeirão Preto, SP Brazil; Department of Pediatrics, School of Medical Sciences, State University of Campinas (UNICAMP), Campinas, SP Brazil; Pediatric Endocrine Unit, Instituto da Criança-Hospital das Clínicas, Universidade de São Paulo (USP), São Paulo, SP Brazil; Pediatrics Department, Medical School/ Hospital das Clínicas, Universidade Federal de Minas Gerais, Belo Horizonte, Brazil

**Keywords:** Hypoglycemia, Neonatal, Congenital

## Abstract

**Objective:**

To study the clinical and molecular characteristics of a sample of Brazilian patients with Congenital Hyperinsulinemic Hypoglycemia (CHH).

**Methods:**

Electronic message was sent to members from Endocrinology Department- Brazilian Society of Pediatrics requesting clinical data for all cases of CHH. A whole blood sample from living patients was requested for DNA extraction followed by a search for mutations of the genes ABCC8, KCNJ11, GCK, GLUD1, HADH, SLC16A1 and HNF4A.

**Results:**

Of the 61 patients evaluated, 36 (59%) were boys, and only 16 (26%) were born by normal delivery. Gestational age ranged from 32 to 41 weeks (mean = 37 weeks and 6 days). Birth weight ranged from 1590 to 5250 g (mean = 3430 g). Macrossomia occurred in 14 cases (28%). Age at diagnosis ranged from 1 to 1080 days (mean = 75 days). DNA for molecular analysis was obtained from 53 of the 61 patients. Molecular changes in the ABCC8 gene were detected in 15 (28%) of these 53 cases, and mutations in the KCNJ11 gene were detected in 6 (11%). Mutations in the GLUD1 gene were detected in 9 cases (17%) of the total series. Mutations of the GCK gene in heterozygosis were detected in 3 cases. No mutations were detected in the sequencing of genes HADH, SLC16A1 and HNF4A.

**Conclusion:**

The present study conducted in Brazil permitted the collaborative compilation of an important number of CHH cases and showed that the present clinical and molecular data are similar to those of published global series.

## Introduction

The maintenance of appropriate blood glucose levels is of vital importance at all ages, but particularly during the neonatal and breastfeeding period. In this phase of life, cerebral tissue is not yet fully mature and is marked by intense metabolic activity and extreme avidity for glucose, being considerably more sensitive to reductions of glycemic levels even if transient. Thus, the occurrence of hypoglycemia in this phase of life is relate to high morbility and mortality [1–4].

Inappropriate insulin secretion in relation to blood glucose levels characterizes hyperinsulinism, which is the main cause of persistent and recurrent hypoglycemia during this phase of life [1, 5, 6].

The congenital hyperinsulinemic hypoglycemia (CHH) represent a heterogeneous group of clinical condition, the most severe and frequent form of HH [6–8]. The CHH may present as different clinical characteristics (responsive or not to diazoxide, elevated or not ammonia levels) and histological subgroups (focal or diffuse form), but the aspect most recently investigated is the genetic of CHH [1–10].

Mutations in seven different genes (*ABCC8*, *KCNJ11*, *GLUD1*, *GCK*, *HADH*, *SLC16A1* and *HNF4A*) are responsible for about 50% of all CHH cases [1, 2]. Of these genes, *ABCC8* and *KCNJ11*, located in regions neighboring chromosome 11 (11p15.1), are responsible for the expression of the proteins SUR1 and Kir6.2. These proteins comprise the potassium channels sensitive to adenosine-triphosphate which play a fundamental role in the control of insulin secretion stimulated by glucose. Inactivating mutations in these two genes are responsible for the most common and severe forms of CHH [9–18]. Defects of the other five genes involved are responsible for a smaller number of cases of CHH [1, 2].

Mutations in these genes are expressed with particular histopathological features, clinical manifestations, therapeutic response to drugs and therefore the investigation of these molecular alterations permits a precise diagnosis and an appropriate management.

The objectives of the present study were to obtain clinical information regarding patients with CHH from the largest possible number of clinical centers in Brazil and to determine the molecular etiology of such cases.

## Patients and methods

An electronic message was sent to all members of the Department of Endocrinology of the Brazilian Society of Pediatrics, involving representatives of almost all Brazilian states, inviting them to participate in this study. Information about all patients with a diagnosis of CHH was requested, such as sex, gestational age, type of delivery, birth weight and length, age at the onset of symptoms, age at diagnosis of the disease, laboratory results, histological form of the disease, response to drug therapy, need of pancreatectomy, presence of sequelae, and mortality.

The study was approved by the Human Research Ethics Committee of the Faculty of Medicine of Ribeirão Preto, University of São Paulo, and the parents or persons responsible for the living patients gave written informed consent to participate. A whole blood sample was then collected from each index case and his/her parents for DNA extraction followed by the determination of mutations of the seven genes.

DNA was extracted from peripheral blood leukocytes using a commercial kit (QIAamp DNA Mini Kit-Qiagen, Dusseldorf, German) and the sample was stored at −70°C; the exon ligands of the intron/exons of the genes were amplified by PCR. The PCR product was then sequenced by a standard method using an ABI 3730 sequencer (Applied Bio systems, Warrington, UK) and the sequences obtained were compared to the published sequences [4–9].

Mutations were first searched in the *ABCC8* and *KCNJ11* genes. When no mutations were detected in these genes, mutations were searched in the *GLUD1* and *GCK* genes and in cases of no mutations, the remaining genes were investigated.

Data are reported as absolute and relative frequency, with means and standard deviations.

## Results

Data and blood samples of 61 children with CHH were obtained. Blood samples were also obtained from the parents of most of these children, for a total of 140 samples.

Most samples were from the Southeast region of Brazil, which concentrates the largest number of pediatric endocrinology centers. However, the Center-West, Northeast and South regions were also represented. The North region did not send samples.

Of the 61 patients evaluated, 36 (59%) were boys. Gestational age ranged from 32 to 41 weeks (mean = 37 and 6 days; SD = 5 days). Birth weight ranged from 1590 to 5250 g (mean = 3430 g; SD = 480 g). Macrossomia occurred in 14 cases (28%). Birth length was available for 42 patients, but this result was excluded because it was found to be unreliable.

Age at diagnosis ranged from 1 to 1080 days (mean = 75 days; SD = 17 days), with a diagnosis being made at more than 90 days of life in 14 (28%) patients.

The diagnosis of HH are suspect by the need of high rate of glucose infusion to maintain normal blood glucose level, and confirmed by the dosage of low blood glucose level, inadequate insulin level, low ketones and free fatty acids levels in serum [1, 2, 19]. Other laboratory tests should de collected at the same time with glucose, insulin, ketones and free fatty acids, as ammonia, lactate and glucagon stimulation test for the diagnostic workout. Except for the glucose and insulin, not all the laboratory tests were carried out for all patients. Blood lactate level was done in 27, ammonia level in 19, ketone level in 17 and free fatty acids in 16 patients. Table 1 lists the results of blood glucose, insulin levels and glucose infusion rate (GIR, mg/kg/min).

**Table 1:** **Blood glucose, insulin levels and glucose infusion rate (GIR) in 61 patients with CHH**

Glucose (mg/dL)	Insulin (IU/mL)	GIR (mg/kg/min)
5-77*	2.5-147*	10-41*
28.5 ± 4.7**	24.9 ± 11.3**	17.2 ± 5.5**

*Range **Median and Standard Deviation.

The initial drug used as treatment was glucorticoid in 40 cases (65%). Treatment with diazoxide failure in 24 patients (40%), who were later, submitted to pancreatectomy. All patients submitted to pancreatectomy presented the histological pattern, performed at the different centers, of the diffuse form of the disease.

Table 2 presents the type of treatment performed and the distribution per patient.

**Table 2:** **Type of drug used as treatment for 61 patients with CHH**

Diazoxide	Glucorticoid	Octreotide	GH*	Nifedipine	Glucagon
41 cases	40 cases	24 cases	14 cases	3 cases	3 cases
67%	66%	39%	23%	5%	5%

*Growth Hormone.

DNA quantity and quality sufficient for molecular analysis was obtained from 53 of the 61 patients (87%). In 15 of these 53 cases (28%), molecular changes were detected in the *ABCC8* gene. All mutations were in heterozygosis, occurring in exon 1 in 6 cases (c.72C > A in 4 and c.134C > T in 2), in exons 31 (c.3992-9G > A) and 36 (c.4415-13G > A) in 2 cases, and in exons 2 (c.257 T > G), 3 (c.331G > A), 4 (c.563A > G), 5 (c.742C > T) and 12 (c.1792C > T) in 1.

Mutations in the *KCNJ11* gene were detected in 6 cases (11%), all of them in heterozygosis, 2 of them being c.801C > G, 2 c.808C > G and 2 c.1142G > A.

The *ABCC8* and *KCNJ11* genes were jointly responsible for mutation in 21 (39%) of 53 cases of CHH.

Analysis of the *GLUD1* gene was carried out in the 32 cases in which no mutations were detected in the *ABCC8* and *KCNJ11* genes. Mutations in this gene were detected in 9 cases (17%), 8 of them being in heterozygosis and 1 in homozygosis. Of these, 7 cases occurred in exon 7 (c.1019A > G), 1 in exon 6 (c.37266C > T) and 1 in exon 10 (c.49322G > T). Serum ammonia was determined in only one of these 9 patients, with a normal result.

Mutations of the *GLUD1* gene were detected in 17% of the total series and in 29% of the cases in which no mutations were detected for the *ABCC8* and *KCNJ11* genes.

The *GCK* gene was analyzed in the 23 cases in which no mutation was detected in the *ABCC8*, *KCNJ11* and *GLUD1* genes. Mutations in heterozygosis were detected in this gene in 3 cases (6%), 2 of them in exon 6 (c.1115C > T) and 1 in exon 10 (c.1829G > T). The *GCK* gene was responsible for 6% of the mutations in the total series and for 10% of the mutations detected in cases that were negative for mutations of the genes associated with the potassium channel.

Sequencing of the *HADH*, *SLC16A1* and *HNF4A* genes did not reveal any mutations.

**Figure 1:**
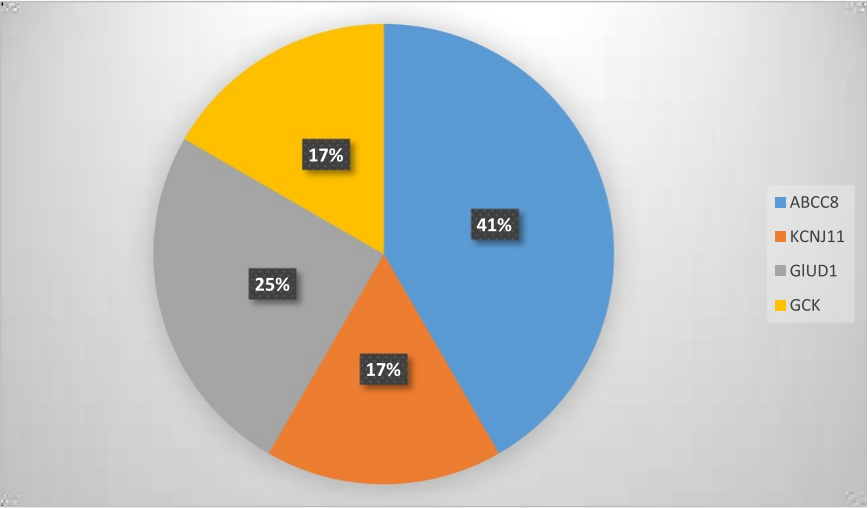
**Distribution by gene of the mutations found in 61 patients with CHH.**

Table 3 and Figure 1 show the distribution of mutations by gene and also their percentages in the group.

**Table 3:** **Mutations found in 61 patients with CHH**

Gene	Exon	Mutation	Number of patients
ABCC8	1	c.72C > A	4
		c.134C > T	2
	2	c.257 T > G	1
	3	c.331G > A	1
	4	c.563A > G	1
	5	c.742C > T	1
	12	c.1792C > T	1
	31	c.3992-9G > A	2
	36	c.4415-13G > A	2
KCNJ11		c.801C > G	2
		c.808C > G	2
		2 c.1142G > A.	2
GLUD1	6	c.37266C > T	1
	7	c.1019A > G	7
	10	c.49322G > T	1
GCK	6	c.1115C > T	2
	10	c.1829G > T	1

The clinical-molecular correlation with the response to diazoxide and with the histological form could not be determined because in 68% of the cases in which diazoxide was used the dose administered was less than 5 mg/kg/d, not considered ideal for the establishment of the response. All those patients that used low dose, didn’t respond to medical treatment.

## Discussion

This was the first large-scale study compiling CHH cases in Brazil. Although most samples were from the Southeast region of the country, all regions were represented, except for the North.

The sample was quite representative in global terms. Recent publications have reported 17 cases for Saudi Arabia [20], 11 cases for China and 33 cases for Italy [21].

The largest samples have been compiled in the two major world reference centers, i.e., England (Great Ormond Hospital) with 300 cases [22] and the United States (Children’s Hospital of Philadelphia) with 417 cases [23] and 223 cases of pancreatectomy [24].

As observed in other series, we also could not detect differences regarding gender and prematurity [20–23].

Birth weight ranged from 1590 to 5250 g, with a mean value of 3430 g. In the present series, macrossomia occurred in 14 cases (28%) as compared to 50% of cases in the Italian sample [21]. The diagnosis was made after 90 days of life in 14 (28%) in our children, later than other series, which is a matter of concern since a delayed diagnosis increases the risk of morbi-mortality.

There is still a substantial heterogeneity regarding the diagnostic threshold for the diagnosis of hypoglycemia in Brazil. Regarding the collection of the diagnostic critical sample, there was no standardization of the exams or of the time when they should be performed. Ammonia was rarely determined, and it should be remembered that hyperammonemia directs the clinical and molecular diagnosis and the clinical conduct regarding the *GLUD1* gene.

There is also a clear need to standardize the treatment, also considering that diazoxide is not a drug easily available in Brazil, requiring importation.

Diazoxide is the medication of choice for the treatment of hypoglycemia due to hyperinsulinism during the neonatal period. None of the other studies have reported the systematic and early use of glucorticoids, as noted in the present series. Nifedipine is not indicated and the use of growth hormone is indicated only in the presence of its deficiency [25–27].

The lack of response to treatment occurred in 40% of the present cases, a much higher rate than reported in the more recent literature [20–24]. Surgery was possibly indicated before the correct use of drug treatment, with appropriate doses. It was interesting to note that only 2 of the most recent 11 cases required pancreatectomy.

It is noteworthy that 100% of the operated cases had the diffuse form of the disease. According to the literature, 1/3 of the operated cases involve the focal form of the disease [22–24] and this difference requires revision of the histological material and clinical-molecular correlation.

Together, genes *ABCC8* and *KCNJ11* were responsible for mutations in 21 of 53 cases (39%) of CHH. This result is similar to obtained in other series, i.e., 47% of cases in the American series [22] and 40.8% in the British one [23].

A clinical-molecular correlation regarding the response to diazoxide and the histological form was found to be impossible.

Thus, mutations of the *GLUD1* gene were detected in 17% of the total series and in 29% of cases in which no mutations of the *ABCC8* and *KCNJ11* genes were detected. As reported earlier, this gene is responsible for the clinical form of hypoglycemia associated with high levels of serum ammonia, and is also the major gene affected in cases in which no mutations are detected in genes ABCC8 and KCNJ11 [28–31]. Serum ammonia was determined in only one of these patients, with a normal result. A high level of serum ammonia directs the molecular investigation and the drug treatment, as the response to diazoxide is usually exuberant, with no low dose. Unfortunately, diazoxide was not used in these 9 cases, a fact that did not permit us to establish the clinical-molecular relationship reported in the literature [19, 28, 32–34].

This result has been observed in large series, with the mutations of this gene being as frequent as those of the genes of the potassium channel in the forms responsive to diazoxide [28–31].

The molecular changes in gene GCK accounted for 6% of the mutations detected in the entire series and for 10% of the mutations detected in the cases that were negative for mutations in the genes associated with the potassium channel, in agreement with previous literature data [35–38].

In general, mutations were detected in 63% of the series, most of them occurring in the two genes coding for the proteins SUR 1 and Kir6.2, responsible for the functioning of the ATP-dependent potassium channel [39].

Thus, the present pioneering study conducted in Brazil permitted the collaborative compilation of an important number of CHH cases from almost all regions of the country, showing that our clinical and molecular data are similar to those for large global series and underscoring the need to standardize the diagnosis and treatment of CHH in our country.

## Abbreviations

CHH: Congenital Hyperinsulinemic Hypoglycemia.
